# Trends in exclusive and dual cigarette and electronic cigarette use among United States middle and high school students from 2017 to 2024: A cross-sectional analysis

**DOI:** 10.18332/tid/231124

**Published:** 2026-10-29

**Authors:** Yixian Chen, Evelyn Jimenez-Mendoza, Jihyoun Jeon, Rafael Meza

**Affiliations:** 1 Department of Population Health SciencesBritish Columbia Cancer Research InstituteVancouverCanada; 2 Department of EpidemiologyUniversity of MichiganAnn ArborUnited States; 3 School of Population and Public Health, University of British ColumbiaVancouverCanada; 4 Rutgers Cancer Institute, Rutgers UniversityNew BrunswickUnited States

**Keywords:** youth tobacco use, survey research, electronic cigarettes, tobacco control, public health

## Abstract

**Introduction:**

The increase in electronic cigarette (e-cigarette) use among United States (US) middle and high school students raises public health concerns. Understanding cigarette and e-cigarette use patterns is crucial for targeted tobacco control policies. This study aimed to identify and characterize changes in trends of exclusive and dual use of cigarettes and e-cigarettes among US middle and high school students from 2017 to 2024, by grade and sex.

**Methods:**

We conducted a secondary analysis of the Monitoring the Future (MTF) survey, an annual, nationally representative cross-sectional survey of US students in Grades 8, 10, and 12 (2017–2024; N=227966). Students self-reported past 30-day cigarette and e-cigarette use via school-administered questionnaires. The survey’s complex sampling design incorporated clustering, stratification, and weighting. We conducted joinpoint regression analyses by grade (8, 10, or 12) and sex (boys or girls).

**Results:**

The prevalence of daily cigarette use in the past 30 days between 2017 and 2024 decreased with annual percent changes (APCs) of -23.3% among Grade 12 boys (95% confidence interval [CI]: -31.7 – -13.9) and -29.5% among girls (95% CI: -35.6 – -22.9). Exclusive cigarette use declined significantly among Grade 12 boys from 2017 to 2024 (APC= -23.0%; 95% CI: -32.8 – -11.6) and girls from 2017 to 2020 (APC= -49.3%; 95% CI: -63.6 – -29.5). E-cigarette use increased sharply until 2019–2020, particularly among girls, then declined significantly mainly among boys; girls’ use remained above 2017 levels (e.g. regular use among Grade 12 girls: average annual percent change [AAPC]=19.7%; 95% CI: 9.6–30.7). From 2017 to 2024, any dual use fell, especially among Grade 10 boys (APC= -16.2%; 95% CI: -22.1 – -9.9) and girls (APC= -14.3%; 95% CI: -18.4 – -9.9).

**Conclusions:**

Cigarette and dual use of cigarettes and e-cigarettes among US middle and high school students declined significantly from 2017 to 2024, suggesting positive shifts in tobacco use behavior, but stable e-cigarette use highlights an ongoing challenge in nicotine control among youth.

## Introduction

E-cigarette use among United States (US) middle and high school students rose after 2017^1,2^, peaking in 2019, with 27.5% of high school and 10.5% of middle school students reporting use^3^. It has since declined, influenced by public health interventions, the outbreak of e-cigarette or vaping product use-associated lung injury (EVALI)^4^, and the COVID-19 pandemic. Cigarette smoking has declined since the mid-1990s, reaching historical lows in 2024: 1.7% among high school and 1.1% among middle school students^5^. Distinguishing between exclusive and dual use is essential, as these patterns have different associated health effects and likelihood of future use^6^. The objective of this study was to identify and characterize changes in trends of exclusive and dual use of cigarettes and e-cigarettes between 2017 and 2024 among US middle and high school students, stratified by grade and sex, to guide public health strategies to prevent youth tobacco use.

## Methods

### Study design and population

The study is a secondary analysis of the Monitoring the Future (MTF) survey^7^, an annual cross-sectional, nationally representative survey of US students in Grades 8, 10, and 12, capturing cigarette and e-cigarette use^8^. Eight waves of annual cross-sectional datasets were analyzed from 2017 to 2024, whose participants were selected through a multistage random sampling method. Students in Grades 8, 10, and 12 who completed an MTF survey in any year from 2017 to 2024 were eligible for inclusion, with no further restrictions applied. Because e-cigarette items were administered on a random subset of questionnaire forms, only students who received those forms were eligible for the analyses of e-cigarette as exclusive use or dual use. The survey initially was self-administered in paper-and-pencil questionnaires in the classrooms, then transitioned to a web-based questionnaire using Internet-connected devices in 2020. The sample size for each year was determined by the MTF survey design, which provides a large sample sufficient for producing precise, nationally representative estimates for the target population of US students. Student response rates within participating schools are generally high; for example, in 2017, the rates were 87%, 85%, and 79% for Grade 8, 10, and 12 students, respectively^9^. After the web-based questionnaires were introduced, the corresponding response rates were 86%, 84%, and 75% in 2022^9^.

### Ethical considerations

This research was carried out utilizing openly accessible, anonymized data and did not need to obtain approval from an Institutional Review Board (IRB). This study followed the Strengthening the Reporting of Observational Studies in Epidemiology (STROBE) guideline for cross-sectional studies^10^; the completed STROBE checklist is provided as the Supplementary file.

### Measures

Middle and high school students reported their cigarette and e-cigarette use within the past 30 days. Cigarette use was measured using the questions: ‘Have you ever smoked cigarettes?’ and ‘How frequently have you smoked cigarettes during the past 30 days?’. E-cigarette use was measured by asking, ‘In your lifetime, how often have you vaped nicotine?’ and ‘On how many days (if any) during the last 30 days have you vaped nicotine?’. E-cigarette-related questions were administered to a random, form-specific subset of the sample. Derived categories for analysis included the following: any cigarette use (≥1 cigarettes), daily cigarette use (≥1 cigarettes per day), exclusive cigarette use (no e-cigarette use), any e-cigarette use (≥1 days), regular e-cigarette use (≥10 days), and exclusive e-cigarette use (no cigarette use). Due to differing measurements, ‘frequent use’ was defined as daily use of cigarettes and regular use of e-cigarettes. Three definitions of dual use were examined: 1) any use of both cigarettes and e-cigarettes (any dual use), 2) daily cigarette use with any e-cigarette use, and 3) any cigarette use with regular e-cigarette use.

### Statistical analysis

Prevalence estimates incorporated survey features (weights, clusters, and strata) and were calculated using R Statistical Software (v4.4.1; R Core Team 2024) to mitigate sampling bias and selection bias. The Joinpoint Regression Program (version 5.4.0)^11,12^ was used to identify significant shifts in the trends, with annual percent change (APC) computed for each segment and average annual percent change (AAPC) summarizing the overall trend throughout the full study period. Participants with missing data on the variables of interest were excluded from analyses. Tests were considered statistically significant at a two-sided p<0.05. Preliminary results were presented at the TCORS Grantee Meeting^13^ and the SRNT annual meeting^14^.

## Results

The study included 227966 students from 2017 to 2024. In the overall sample there were: 75044 Grade 8; 82818 Grade 10; and 70104 Grade 12 students. Over eight years, 114001 boys and 113965 girls participated in the study. The number of students changed over time, with a decrease in 2020 (n=11516). To provide transparency regarding estimate stability, annual unweighted sample sizes and unweighted percentages for all cigarette and e-cigarette use variables are provided in Table 1. Table 2 summarizes the weighted prevalence of past 30-day cigarette and e-cigarette use by grade. Prevalence trends are presented in Figures 1–3. Joinpoint regression results (APCs and AAPCs) by grade and sex are presented in Table 3.

**Table 1:** Characteristics of the participants and past 30-day cigarette and e-cigarette use measures among students in Grades 8, 10, and 12; Monitoring the Future cross-sectional survey, United States, 2017–2024 (N=227966)

Characteristics	2017	2018	2019	2020	2021	2022	2023	2024
**Grade**								
8	14460 (36.5)	13167 (33.4)	13273 (33.5)	3104 (27.0)	10067 (35.5)	8606 (31.5)	5611 (28.0)	6756 (30.7)
10	13143 (33.2)	13888 (35.2)	13857 (35.0)	4805 (41.7)	10373 (36.5)	10293 (37.6)	7603 (37.9)	8856 (40.3)
12	11963 (30.2)	12394 (31.4)	12484 (31.5)	3607 (31.3)	7957 (28.0)	8462 (30.9)	6851 (34.1)	6386 (29.0)
**Sex**								
Boys	19508 (49.3)	19454 (49.3)	19624 (49.5)	5622 (48.8)	14368 (50.6)	14192 (51.9)	10150 (50.6)	11083 (50.4)
Girls	20058 (50.7)	19995 (50.7)	19990 (50.5)	5894 (51.2)	14029 (49.4)	13169 (48.1)	9915 (49.4)	10915 (49.6)
**Any cigarette**								
Yes	2055 (5.2)	1688 (4.3)	1319 (3.3)	383 (3.3)	498 (1.8)	475 (1.7)	377 (1.9)	334 (1.5)
No	37511 (94.8)	37761 (95.7)	38295 (96.7)	11133 (96.7)	27899 (98.2)	26886 (98.3)	19688 (98.1)	21664 (98.5)
**Daily cigarette**								
Yes	825 (2.1)	670 (1.7)	496 (1.3)	141 (1.2)	168 (0.6)	178 (0.7)	106 (0.5)	72 (0.3)
No	38741 (97.9)	38779 (98.3)	39118 (98.7)	11375 (98.8)	28229 (99.4)	27183 (99.3)	19959 (99.5)	21926 (99.7)
**Exclusive cigarette**								
Yes	344 (2.7)	182 (1.5)	204 (0.8)	47 (0.7)	118 (0.4)	135 (0.5)	104 (0.5)	78 (0.4)
No	12298 (97.3)	12318 (98.5)	24945 (99.2)	6354 (99.3)	28227 (99.6)	27158 (99.5)	19918 (99.5)	21869 (99.6)
**Any e-cigarette**								
Yes	998 (7.7)	1971 (15.3)	4809 (18.9)	1130 (17.6)	3578 (12.6)	3646 (13.3)	2567 (12.8)	2211 (10.1)
No	11929 (92.3)	10904 (84.7)	20622 (81.1)	5278 (82.4)	24789 (87.4)	23669 (86.7)	17471 (87.2)	19750 (89.9)
**Regular e-cigarette**								
Yes	315 (2.4)	855 (6.6)	2262 (8.9)	449 (7.0)	1611 (5.7)	1752 (6.4)	1243 (6.2)	1118 (5.1)
No	12612 (97.6)	12020 (93.4)	23169 (91.1)	5959 (93.0)	26756 (94.3)	25563 (93.6)	18795 (93.8)	20843 (94.9)
**Exclusive e-cigarette**								
Yes	675 (5.3)	1582 (12.7)	4155 (16.5)	975 (15.2)	3196 (11.3)	3306 (12.1)	2294 (11.5)	1952 (8.9)
No	11967 (94.7)	10918 (87.3)	20994 (83.5)	5426 (84.8)	25149 (88.7)	23987 (87.9)	17728 (88.5)	19995 (91.1)
**Any dual**								
Yes	302 (2.4)	313 (2.5)	598 (2.4)	153 (2.4)	378 (1.3)	338 (1.2)	270 (1.3)	253 (1.2)
No	12340 (97.6)	12187 (97.5)	24551 (97.6)	6248 (97.6)	27967 (98.7)	26955 (98.8)	19752 (98.7)	21694 (98.8)
**Daily cigarette and any e-cigarette**								
Yes	125 (1.0)	117 (0.9)	226 (0.9)	56 (0.9)	113 (0.4)	102 (0.4)	57 (0.3)	53 (0.2)
No	12517 (99.0)	12383 (99.1)	24923 (99.1)	6345 (99.1)	28232 (99.6)	27191 (99.6)	19965 (99.7)	21894 (99.8)
**Any cigarette and regular e-cigarette**								
Yes	133 (1.1)	198 (1.6)	404 (1.6)	92 (1.4)	252 (0.9)	228 (0.8)	184 (0.9)	172 (0.8)
No	12509 (98.9)	12302 (98.4)	24745 (98.4)	6309 (98.6)	28093 (99.1)	27065 (99.2)	19838 (99.1)	21775 (99.2)

Data are given as unweighted sample sizes and unweighted column percentages, n (%). For grade and sex, the denominator is the total sample in that year; for each use measure, the denominator is the number of students with a valid response to that measure in that year. Unweighted percentages describe the analytic sample and are not prevalence estimates; weighted prevalence estimates are presented in Table 2. E-cigarette: electronic cigarette. All measures are self-reported and refer to the past 30 days. Any cigarette: smoked ≥1 cigarettes. Daily cigarette: smoked ≥1 cigarettes per day. Exclusive cigarette: any cigarette use with no e-cigarette use. Any e-cigarette: vaped nicotine on ≥1 days. Regular e-cigarette: vaped nicotine on ≥10 days. Exclusive e-cigarette: any e-cigarette use with no cigarette use. Any dual use: any cigarette use and any e-cigarette use. Daily cigarette and any e-cigarette: daily cigarette use with any e-cigarette use. Any cigarette and regular e-cigarette: any cigarette use with regular e-cigarette use. Measures involving both products (exclusive and dual use) require valid responses to both the cigarette and e-cigarette questions.

**Table 2:** Weighted prevalence of past 30-day cigarette and e-cigarette use among students in Grades 8, 10, and 12; Monitoring the Future cross-sectional survey, United States, 2017–2024 (N=227966)

Year	Grade 8	Grade 10	Grade 12	Grade 8	Grade 10	Grade 12	Grade 8	Grade 10	Grade 12
	% (95% CI)	% (95% CI)	% (95% CI)	% (95% CI)	% (95% CI)	% (95% CI)	% (95% CI)	% (95% CI)	% (95% CI)
	**Any cigarette**			**Daily cigarette**			**Exclusive cigarette**		
2017	1.9 (1.5–2.4)	4.9 (4.3–5.5)	9.5 (8.1–11.1)	0.6 (0.4–0.7)	2.1 (1.7–2.6)	4.1 (3.3–5.0)	0.9 (0.6–1.5)	2.8 (2.3–3.4)	4.9 (3.8–6.2)
2018	2.1 (1.8–2.4)	4.1 (3.2–5.2)	7.0 (5.5–8.9)	0.8 (0.5–1.1)	1.7 (1.1–2.6)	3.1 (2.2–4.5)	1.1 (0.6–1.8)	1.2 (0.7–1.8)	3.2 (2.1–4.8)
2019	2.2 (1.6–3.0)	3.3 (2.6–4.2)	5.4 (4.4–6.6)	0.7 (0.5–1.1)	1.3 (1.0–1.8)	2.2 (1.6–2.9)	0.7 (0.3–1.4)	0.6 (0.4–0.9)	1.6 (1.1–2.3)
2020	2.1 (1.4–3.1)	3.1 (2.2–4.2)	6.9 (3.5–13.2)	0.7 (0.4–1.4)	1.1 (0.8–1.4)	2.7 (1.4–5.1)	0.3 (0.2–0.6)	0.7 (0.4–1.2)	1.1 (0.4–2.6)
2021	1.0 (0.7–1.4)	1.4 (1.0–2.1)	3.3 (2.1–5.2)	0.3 (0.1–0.7)	0.5 (0.3–0.9)	1.4 (0.7–2.6)	0.3 (0.2–0.4)	0.2 (0.2–0.4)	0.8 (0.5–1.1)
2022	0.6 (0.4–0.9)	1.5 (1.0–2.2)	3.7 (3.0–4.5)	0.3 (0.2–0.4)	0.6 (0.4–0.7)	1.4 (1.0–1.9)	0.2 (0.1–0.4)	0.3 (0.2–0.6)	0.9 (0.6–1.5)
2023	1.0 (0.6–1.6)	2.0 (1.2–3.2)	2.5 (1.9–3.2)	0.3 (0.2–0.7)	0.7 (0.4–1.2)	0.5 (0.3–0.9)	0.4 (0.3–0.7)	0.8 (0.5–1.1)	0.7 (0.5–1.0)
2024	0.5 (0.4–0.7)	1.3 (1.0–1.6)	2.5 (1.7–3.5)	0.1 (0.1–0.3)	0.3 (0.2–0.5)	0.4 (0.3–0.7)	0.1 (0.1–0.3)	0.3 (0.3–0.5)	0.6 (0.4–0.9)
	**Any e-cigarette**			**Regular e-cigarette**			**Exclusive e-cigarette**		
2017	3.6 (2.8–4.5)	8.2 (6.5–10.3)	11.3 (9.6–13.3)	0.5 (0.2–1.1)	2.6 (1.7–4.0)	3.9 (3.0–5.0)	2.6 (2.0–3.5)	5.5 (4.3–7.1)	7.1 (5.7–8.8)
2018	6.1 (4.8–7.8)	16.0 (13.3–19.2)	21.1 (17.7–25.1)	1.5 (1.1–2.1)	6.5 (4.9–8.5)	10.0 (8.1–12.3)	5.2 (3.9–6.8)	13.3 (10.5–16.7)	17.1 (14.0–20.9)
2019	9.6 (8.1–11.2)	20.5 (18.5–22.6)	25.4 (23.0–28.1)	2.8 (2.3–3.4)	9.1 (7.6–10.9)	14.0 (11.8–16.6)	8.1 (7.0–9.3)	18.2 (16.5–20.0)	21.6 (19.1–24.2)
2020	10.8 (7.5–15.2)	21.4 (16.4–27.5)	22.0 (15.9–29.5)	3.8 (2.1–6.7)	9.4 (6.3–13.7)	12.0 (8.3–17.0)	8.9 (6.2–12.5)	18.9 (13.9–25.1)	17.1 (11.2–25.4)
2021	7.0 (5.2–9.6)	13.0 (10.6–15.8)	19.2 (15.7–23.3)	2.5 (1.6–3.8)	5.8 (4.6–7.2)	9.6 (7.2–12.7)	6.3 (4.6–8.8)	11.8 (9.6–14.4)	16.6 (13.5–20.4)
2022	6.6 (4.7–9.4)	14.0 (11.6–16.8)	20.9 (17.9–24.3)	2.8 (1.8–4.2)	6.0 (4.7–7.6)	11.2 (8.9–13.9)	6.2 (4.4–8.7)	12.8 (10.3–15.8)	18.1 (15.3–21.4)
2023	6.6 (4.4–9.9)	11.4 (9.4–13.8)	17.2 (13.6–21.4)	2.5 (1.3–4.5)	4.7 (3.4–6.3)	9.5 (6.7–13.1)	6.1 (4.1–9.0)	10.2 (8.5–12.2)	15.5 (11.9–19.8)
2024	5.4 (4.2–6.9)	9.3 (7.6–11.3)	14.7 (12.7–17.0)	2.0 (1.5–2.7)	4.3 (3.2–5.8)	8.6 (7.1–10.3)	5.0 (3.9–6.4)	8.4 (6.7–10.4)	12.8 (11.3–14.5)
	**Any dual**			**Daily cigarette and any e-cigarette**			**Any cigarette and regular e-cigarette**		
2017	0.9 (0.5–1.6)	2.6 (2.0–3.4)	4.3 (3.4–5.4)	0.2 (0.1–0.4)	1.3 (0.9–1.9)	1.7 (1.4–2.1)	0.2 (0.1–0.3)	1.2 (0.8–1.9)	1.9 (1.4–2.7)
2018	0.8 (0.7–1.0)	2.5 (1.9–3.3)	3.8 (2.7–5.5)	0.2 (0.1–0.5)	1.0 (0.6–1.7)	1.6 (0.8–3.1)	0.4 (0.2–0.6)	1.5 (1.0–2.1)	2.6 (1.7–3.9)
2019	1.4 (1.0–2.1)	2.4 (1.9–3.1)	3.9 (3.1–4.8)	0.6 (0.3–1.0)	1.1 (0.7–1.7)	1.5 (1.0–2.0)	0.8 (0.5–1.2)	1.6 (1.2–2.1)	2.8 (2.3–3.4)
2020	1.8 (1.2–2.8)	2.6 (1.5–4.4)	4.9 (2.4–9.8)	0.7 (0.3–1.8)	0.7 (0.4–1.2)	2.4 (1.0–5.7)	1.1 (0.6–1.8)	1.7 (0.8–3.5)	3.7 (1.5–8.6)
2021	0.7 (0.4–1.1)	1.2 (0.8–1.8)	2.5 (1.5–4.2)	0.2 (0.1–0.5)	0.4 (0.2–0.7)	0.9 (0.5–1.8)	0.4 (0.2–0.7)	0.8 (0.5–1.4)	1.8 (1.0–3.3)
2022	0.4 (0.3–0.7)	1.2 (0.7–1.9)	2.8 (2.2–3.5)	0.1 (0.1–0.3)	0.4 (0.2–0.6)	0.9 (0.6–1.4)	0.3 (0.2–0.5)	0.7 (0.5–1.1)	2.0 (1.6–2.5)
2023	0.5 (0.2–1.0)	1.2 (0.6–2.3)	1.7 (1.2–2.4)	0.1 (0.0–0.3)	0.4 (0.2–0.6)	0.3 (0.2–0.5)	0.3 (0.1–0.9)	0.8 (0.4–1.5)	1.2 (0.8–1.7)
2024	0.4 (0.2–0.6)	0.9 (0.6–1.3)	1.9 (1.3–2.7)	0.1 (0.1–0.3)	0.2 (0.2–0.3)	0.3 (0.1–0.6)	0.2 (0.1–0.4)	0.6 (0.5–0.9)	1.2 (0.8–1.9)

CI: confidence interval. E-cigarette: electronic cigarette. All measures are self-reported and refer to the past 30 days. Any cigarette: smoked ≥1 cigarettes. Daily cigarette: smoked ≥1 cigarettes per day. Exclusive cigarette: any cigarette use with no e-cigarette use. Any e-cigarette: vaped nicotine on ≥1 days. Regular e-cigarette: vaped nicotine on ≥10 days. Exclusive e-cigarette: any e-cigarette use with no cigarette use. Any dual use: any cigarette use and any e-cigarette use. Daily cigarette and any e-cigarette: daily cigarette use with any e-cigarette use. Any cigarette and regular e-cigarette: any cigarette use with regular e-cigarette use. Measures involving both products (exclusive and dual use) require valid responses to both the cigarette and e-cigarette questions.

**Figure 1:**
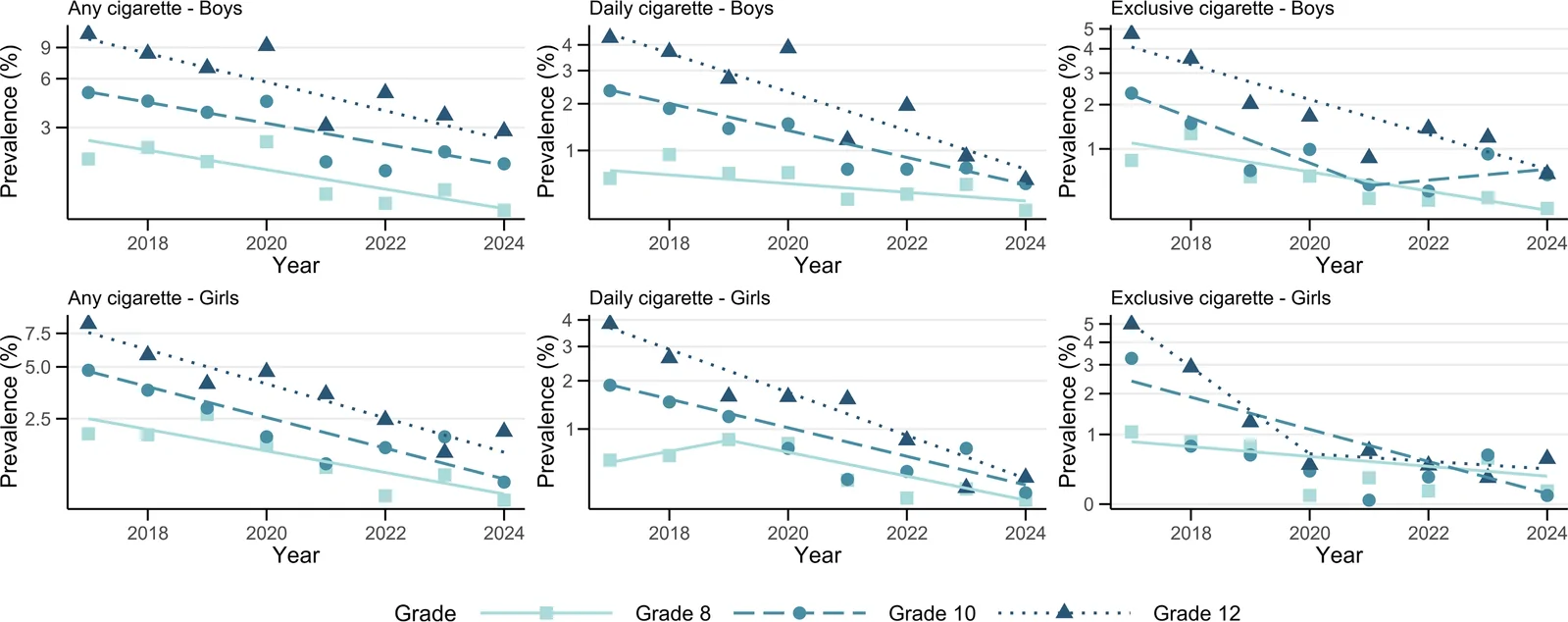
Weighted prevalence and trends in past 30-day any, daily, and exclusive cigarette use among US students in Grades 8, 10, and 12, by sex

E-cigarette: electronic cigarette. All measures are self-reported and refer to the past 30 days. Any cigarette: smoked ≥1 cigarette. Daily cigarette: smoked ≥1 cigarette per day. Exclusive cigarette: any cigarette use with no e-cigarette use. E-cigarette questions were administered to a random form-specific subset of respondents; exclusive cigarette use is estimated within this subset.

**Figure 2:**
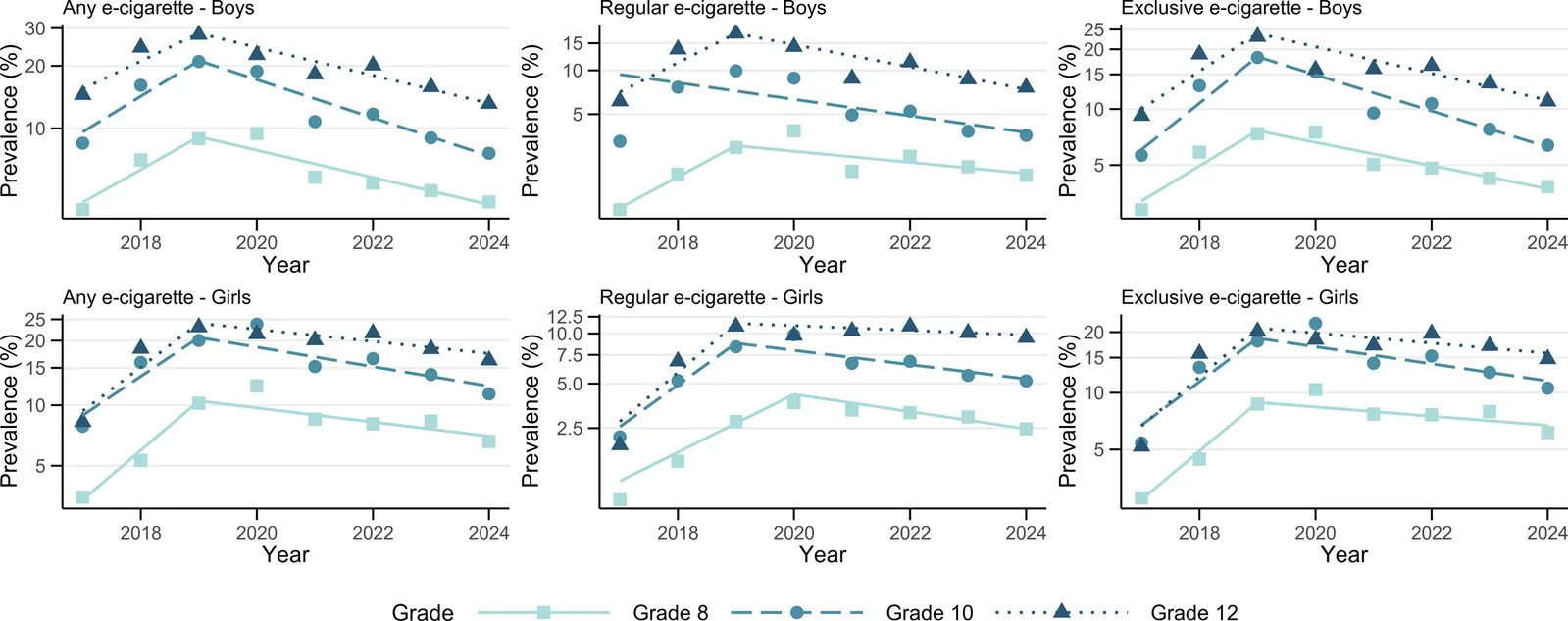
Weighted prevalence and trends in past 30-day any, regular, and exclusive e-cigarette use among US students in Grades 8, 10, and 12, by sex

E-cigarette, electronic cigarette. Dots represent the weighted annual prevalence estimates, with lines indicating the best-fitting joinpoint models. Data are plotted on a logarithmic scale. Prevalence scales vary across panels. All measures are self-reported and refer to the past 30 days. Any e-cigarette: vaped nicotine on ≥1 days. Regular e-cigarette: vaped nicotine on ≥10 days. Exclusive e-cigarette: any e-cigarette use with no cigarette use. E-cigarette questions were administered to a random form-specific subset of respondents.

**Figure 3:**
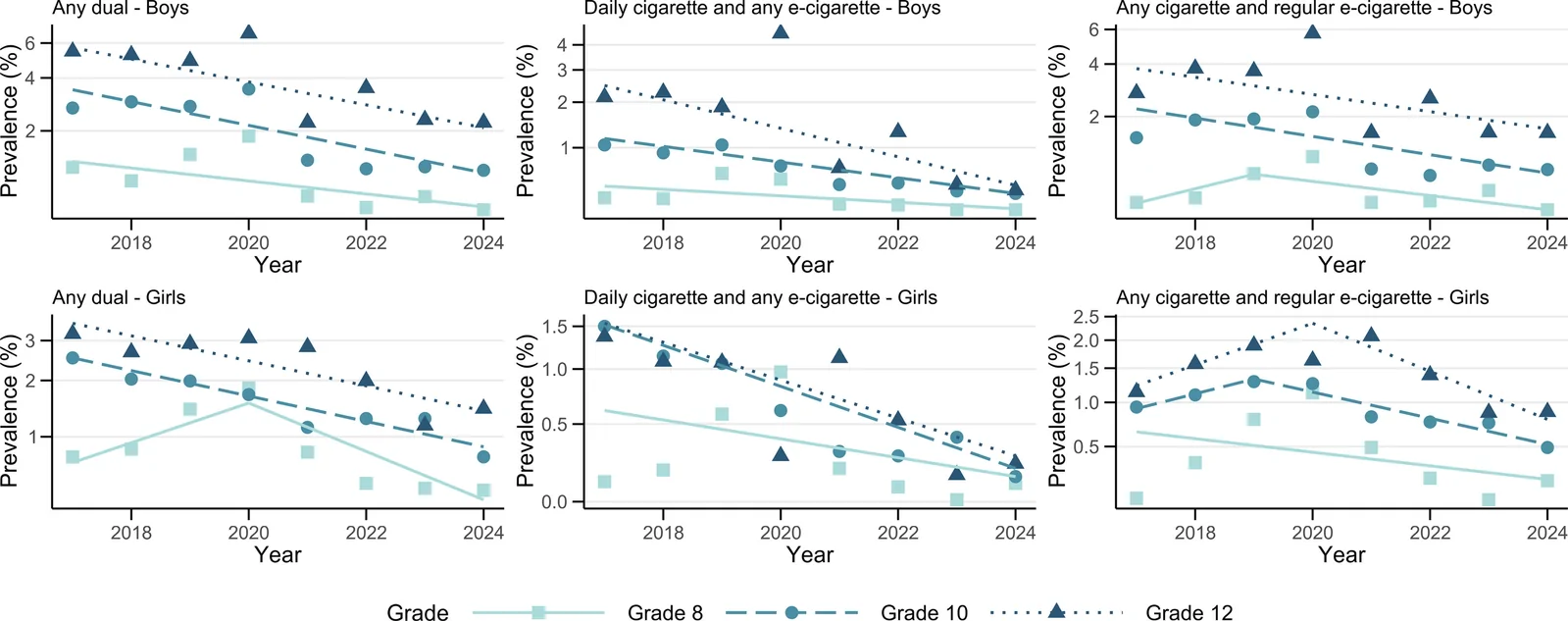
Weighted prevalence and trends in past 30-day any dual, daily cigarette and any e-cigarette, and any cigarette and regular e-cigarette use among US students in Grades 8, 10, and 12, by sex

E-cigarette, electronic cigarette. Dots represent the weighted annual prevalence estimates, with lines indicating the best-fitting joinpoint models. Data are plotted on a logarithmic scale. Prevalence scales vary across panels. All measures are self-reported and refer to the past 30 days. Any dual use: any cigarette use and any e-cigarette use. Daily cigarette and any e-cigarette: daily cigarette use with any e-cigarette use. Any cigarette and regular e-cigarette: any cigarette use with regular e-cigarette use. E-cigarette questions were administered to a random form-specific subset of respondents.

**Table 3:** Annual percentage change (APC) and average annual percentage change (AAPC), based on joinpoint regression, for past 30-day exclusive and dual use of cigarettes and e-cigarettes among students in Grades 8, 10, and 12, by sex; Monitoring the Future cross-sectional survey, United States, 2017–2024 (N=227966)

Grade	Sex	Trend 1		Trend 2		AAPC (95% CI) 2017–2024
		Years	APC (95%CI)	Years	APC (95%CI)	
Any cigarette						
8	Boys	2017–2024	-18.7 (−27.6 – -8.6)*			-18.7 (−27.6 – -8.6)*
8	Girls	2017–2024	-18.5 (−27.2 – -8.8)*			-18.5 (−27.2 – -8.8)*
10	Boys	2017–2024	-15.1 (−19.7 – -10.2)*			-15.1 (−19.7 – -10.2)*
10	Girls	2017–2024	-21.5 (−26.0 – -16.7)*			-21.5 (−26.0 – -16.7)*
12	Boys	2017–2024	-18.0 (−25.9 – -9.3)*			-18.0 (−25.9 – -9.3)*
12	Girls	2017–2024	-20.8 (−25.8 – -15.6)*			-20.8 (−25.8 – -15.6)*
Daily cigarette						
8	Boys	2017–2024	-11.4 (−21.6–0.2)			-11.4 (−21.6–0.2)
8	Girls	2017–2019	26.1 (−36.4–149.7)	2019–2024	-34.4 (−56.7 – -0.6)*	-20.9 (−36.5 – -1.6)*
10	Boys	2017–2024	-20.2 (−24.5 – -15.7)*			-20.2 (−24.5 – -15.7)*
10	Girls	2017–2024	-25.3 (−33.1 – -16.5)*			-25.3 (−33.1 – -16.5)*
12	Boys	2017–2024	-23.3 (−31.7 – -13.9)*			-23.3 (−31.7 – -13.9)*
12	Girls	2017–2024	-29.5 (−35.6 – -22.9)*			-29.5 (−35.6 – -22.9)*
Exclusive cigarette						
8	Boys	2017–2024	-25.7 (−35.3 – -14.7)*			-25.7 (−35.3 – -14.7)*
8	Girls	2017–2024	-13.1 (−30.6–8.8)			-13.1 (−30.6–8.8)
10	Boys	2017–2021	-34.1 (−62.7–16.6)	2021–2024	15.7 (−53.3–186.7)	-16.1 (−38.6–14.7)
10	Girls	2017–2024	-35.6 (−52.9 – -12.0)*			-35.6 (−52.9 – -12.0)*
12	Boys	2017–2024	-23.0 (−32.8 – -11.6)*			-23.0 (−32.8 – -11.6)*
12	Girls	2017–2020	-49.3 (−63.6 – -29.5)*	2020–2024	-10.6 (−43.8–42.1)	-29.9 (−41.7 – -15.7)*
Any e-cigarette						
8	Boys	2017–2019	48.8 (0.1–121.1)*	2019–2024	-15.1 (−24.8 – -4.3)*	-0.4 (−8.7–8.7)
8	Girls	2017–2019	79.2 (7.3–199.2)*	2019–2024	-7.6 (−17.9–4.0)	11.7 (0.6–23.9)*
10	Boys	2017–2019	48.4 (−27.5–203.8)	2019–2024	-19.2 (−30.7 – -5.9)*	-3.9 (−16.7–10.8)
10	Girls	2017–2019	52.2 (−12.8–165.6)	2019–2024	-9.9 (−21.5–3.6)	4.7 (−6.7–17.5)
12	Boys	2017–2019	35.1 (−5.5–93.2)	2019–2024	-14.1 (−23.2 – -4.0)*	-2.3 (−9.8–5.9)
12	Girls	2017–2019	59.9 (1.5–151.8)*	2019–2024	-6.1 (−15.2–3.8)	9.3 (−0.3–19.7)
Regular e-cigarette						
8	Boys	2017–2019	111.6 (−4.1–366.7)	2019–2024	-10.6 (−28.7–12.0)	14.3 (−3.6–35.6)
8	Girls	2017–2020	72.0 (43.5–106.2)*	2020–2024	-12.8 (−20.0 – -5.0)*	16.7 (10.3–23.5)*
10	Boys	2017–2024	-12.6 (−23.7–0.2)			-12.6 (−23.7–0.2)
10	Girls	2017–2019	85.5 (−8.1–274.2)	2019–2024	-9.4 (−20.2–2.8)	11.1 (−2.9–27.3)
12	Boys	2017–2019	55.6 (−14.1–182.1)	2019–2024	-15.5 (−26.9 – -2.4)*	0.6 (−11.0–13.7)
12	Girls	2017–2019	102.8 (29.1–218.6)*	2019–2024	-3.1 (−10.9–5.5)	19.7 (9.6–30.7)*
Exclusive e-cigarette						
8	Boys	2017–2019	58.7 (18.9–111.8)*	2019–2024	-13.9 (−20.9 – -6.3)*	2.5 (−3.8–9.2)
8	Girls	2017–2019	88.7 (10.8–221.3)*	2019–2024	-5.4 (−14.6–4.8)	15.2 (3.9–27.9)*
10	Boys	2017–2019	73.7 (−2.9–210.5)	2019–2024	-19.3 (−29.8 – -7.3)*	0.5 (−10.8–13.2)
10	Girls	2017–2019	66.0 (−24.5–264.9)	2019–2024	-9.3 (−21.0–4.2)	7.8 (−7.3–25.4)
12	Boys	2017–2019	54.7 (10.0–117.7)*	2019–2024	-14.3 (−22.8 – -4.8)*	1.5 (−5.9–9.5)
12	Girls	2017–2019	76.7 (−13.0–259.0)	2019–2024	-5.5 (−16.0–6.3)	13.0 (−1.3–29.3)
Any dual						
8	Boys	2017–2024	-13.7 (−25.8–0.2)			-13.7 (−25.8–0.2)
8	Girls	2017–2020	33.1 (−15.4–109.3)	2020–2024	-35.2 (−52.1– -12.4)*	-11.8 (−24.8–3.5)
10	Boys	2017–2024	-16.2 (−22.1 – -9.9)*			-16.2 (−22.1 – -9.9)*
10	Girls	2017–2024	-14.3 (−18.4 – -9.9)*			-14.3 (−18.4 – -9.9)*
12	Boys	2017–2024	-13.4 (−20.5 – -5.6)*			-13.4 (−20.5 – -5.6)*
12	Girls	2017–2024	-12.2 (−17.9 – -6.2)*			-12.2 (−17.9 – -6.2)*
Daily cigarette and any e-cigarette						
8	Boys	2017–2024	-12.8 (−26.6–3.6)			-12.8 (−26.6–3.6)
8	Girls	2017–2024	-19.2 (−51.3–33.9)			-19.2 (−51.3–33.9)
10	Boys	2017–2024	-16.7 (−21.7 – -11.3)*			-16.7 (−21.7 – -11.3)*
10	Girls	2017–2024	-25.8 (−33.8 – -16.7)*			-25.8 (−33.8 – -16.7)*
12	Boys	2017–2024	-22.2 (−33.6 – -8.8)*			-22.2 (−33.6 – -8.8)*
12	Girls	2017–2024	-22.0 (−32.3 – -10.2)*			-22.0 (−32.3 – -10.2)*
Any cigarette and regular e-cigarette						
8	Boys	2017–2019	56.7 (−78.9–1065.0)	2019–2024	-21.4 (−47.9–18.5)	-4.3 (−35.6–42.3)
8	Girls	2017–2024	-15.0 (−41.1–22.5)			-15.0 (−41.1–22.5)
10	Boys	2017–2024	-14.7 (−22.4 – -6.2)*			-14.7 (−22.4 – -6.2)*
10	Girls	2017–2019	19.9 (−43.2–153.2)	2019–2024	-17.1 (−27.3 – -5.6)*	-7.9 (−20.2–6.3)
12	Boys	2017–2024	-11.1 (−20.1 – -1.0)*			-11.1 (−20.1 – -1.0)*
12	Girls	2017–2020	24.2 (−3.0–59.1)	2020–2024	-23.9 (−39.4 – -4.5)*	-6.2 (−15.4–4.1)

CI: confidence interval. E-cigarette: electronic cigarette. All measures are self-reported and refer to the past 30 days. Any cigarette: smoked ≥1 cigarettes. Daily cigarette: smoked ≥1 cigarettes per day. Exclusive cigarette: any cigarette use with no e-cigarette use. Any e-cigarette: vaped nicotine on ≥1 days. Regular e-cigarette: vaped nicotine on ≥10 days. Exclusive e-cigarette: any e-cigarette use with no cigarette use. Any dual use: any cigarette use and any e-cigarette use. Daily cigarette and any e-cigarette: daily cigarette use with any e-cigarette use. Any cigarette and regular e-cigarette: any cigarette use with regular e-cigarette use. Measures involving both products (exclusive and dual use) require valid responses to both the cigarette and e-cigarette questions.

* Annual percent change (APC) and average annual percent change (AAPC) estimated using joinpoint regression are significantly different from zero, p<0.05.

### Trends in cigarette use by sex and grade

Any cigarette use declined in all grade and sex groups from 2017 to 2024, with no joinpoints detected and APCs ranging from -15.1% to -21.5% (Table 3, Figure 1). Declines occurred in both sexes but were steeper among girls in Grades 10 and 12. Daily cigarette use fell even faster among Grade 10 and 12 students (APCs from -20.2% to -29.5%), whereas in Grade 8, the decline among boys was not statistically significant, and girls’ use declined significantly only after 2019 (APC= -34.4%; 95% CI: -56.7 – -0.6). Exclusive cigarette use showed the strongest reductions of the three cigarette outcomes. Grade 8 boys, Grade 10 girls, and Grade 12 boys declined steadily from 2017 to 2024 (APCs from -23.0% to -35.6%), and Grade 12 girls’ prevalence dropped by nearly half annually from 2017 to 2020 (APC= -49.3%; 95% CI: -63.6 – -29.5) before leveling off. Trends among Grade 8 girls and Grade 10 boys did not reach statistical significance.

### Trends in e-cigarette use by sex and grade

In contrast with the steady declines in cigarette use, e-cigarette use rose and fell. Prevalence increased from 2017 to a peak around 2019, then declined or stabilized through 2024 in nearly every group (Table 3, Figure 2). The initial increases were sharpest among girls, most markedly for regular e-cigarette use among Grade 12 girls (APC=102.8%, 95% CI: 29.1–218.6; during 2017–2019) and Grade 8 girls (APC=72.0%; 95% CI: 43.5–106.2; during 2017–2020). The subsequent declines, however, reached statistical significance almost exclusively among boys, whose any and exclusive e-cigarette use fell by 13.9% to 19.3% annually after 2019 in all three grades; among girls, the only significant post-peak decline was in regular use among Grade 8 girls (APC= -12.8%; 95% CI: -20.0 – -5.0). Consequently, over the full 2017–2024 period, AAPCs were significantly positive only among girls (AAPCs of 11.7%, 16.7%, and 15.2% for any, regular, and exclusive use in Grade 8 girls, respectively, and AAPC of 19.7% for regular use in Grade 12 girls), indicating that girls’ e-cigarette use remained above 2017 levels while boys’ use returned toward baseline.

### Trends in dual use by sex and grade

Dual use declined under all three definitions, with significant reductions concentrated among Grade 10 and 12 students (Table 3, Figure 3). For any dual use and for daily cigarette use with any e-cigarette use, boys and girls in both grades declined significantly across the full 2017–2024 period (APCs from -12.2% to -25.8%). For any cigarette with regular e-cigarette use, boys again declined steadily during 2017–2024, whereas girls’ declines emerged only after peaks in 2019 for Grade 10 (APC= -17.1%; 95% CI: -27.3 – -5.6) and 2020 for Grade 12 (APC= -23.9%; 95% CI: -39.4 – -4.5). Grade 8 students’ trends were mostly non-significant; the exception was a sharp decline in any dual use among Grade 8 girls after 2020 (APC= -35.2%; 95% CI: -52.1 – -12.4).

## Discussion

Exclusive cigarette use and dual use declined among US middle and high school students, with cigarette use reaching historical lows. These patterns are consistent with the National Youth Tobacco Survey (NYTS), in which cigarette smoking fell in 2024 to the lowest prevalence recorded, and e-cigarette use declined further between 2023 and 2024^5^. A joinpoint analysis of NYTS data from 2013 to 2022 similarly identified 2019 as the peak of youth e-cigarette use, followed by declines^15^. E-cigarette use has risen but has declined recently. Any, regular, and exclusive e-cigarette use trends initially increased, particularly among girls, before stabilizing. This stabilization among middle and high school students differs from the significant, ongoing increase in non-combustible product use observed in US adults from 2017 to 2023^16^. Grade 8 boys and girls, and Grade 12 boys, showed increases in exclusive e-cigarette use from 2017 to 2019. These patterns may reflect shifting social norms, with cigarette smoking seen as less acceptable and e-cigarette use perceived as more acceptable or less harmful^17^.

Although definitions of dual use varied, all showed declining trends, particularly among boys and girls in Grades 10 and 12, indicating progress in reducing co-use. These trends suggest fewer middle and high school students are engaging in the co-use of these products, potentially reducing their exposure to harmful substances^18^. In the NYTS data covering 2014 to 2020, a window spanning the rise of youth vaping, dual cigarette and e-cigarette use increased more among middle school than high school students^19^. Our results extend this literature through 2024 and indicate that dual use subsequently declined under all three definitions, with reductions concentrated in Grades 10 and 12.

Given the nationally representative sample of US students in Grades 8, 10, and 12, the results of this study are generalizable to the broader US middle and high school student populations in the surveyed grades. Sex- and grade-specific differences in cigarette and e-cigarette use trends may highlight the importance of continued surveillance to guide prevention strategies. Grade 12 girls showed stronger increases in any and regular e-cigarette use than boys from 2017 to 2019. The sex divergence we observed mirrors national patterns: in NYTS, e-cigarette use among high school girls exceeded that among boys in 2023^20^, and girls accounted for over half of students who had ever used e-cigarettes by 2022^15^.

### Limitations

Several limitations of this study should be acknowledged. First, although this study described and characterized patterns of exclusive and dual cigarette and e-cigarette use, the survey’s cross-sectional design limits causal inference. Second, our study period (2017–2024) coincided with notable regulatory and public health events, including JUUL’s rise and fall, the 2020 federal enforcement policy prioritizing action against flavored cartridge-based e-cigarettes, and Tobacco 21 legislation^21,22^; these historical shifts may confound interpretation of the observed trends independent of any underlying behavior change. Because that policy exempted tobacco- and menthol-flavored products and did not cover disposable devices^22^, it may have shifted the types of products used rather than overall prevalence. Third, methodological changes and lower response rates during the COVID-19 pandemic may affect the comparability of 2020 data^23^. Fourth, it is possible for students surveyed in an earlier wave (e.g. Grade 8 in 2017) to be re-surveyed in later waves (e.g. Grade 10 in 2019 and Grade 12 in 2021). As the data are anonymized, we could not identify these overlapping participants, and each survey wave was therefore treated as an independent sample. Fifth, because cigarette and e-cigarette use were measured using different frequency thresholds (daily use for cigarettes vs use on ≥10 days for e-cigarettes), the ‘frequent use’ thresholds for cigarettes and e-cigarettes are not directly comparable. This highlights the need for standardized definitions of frequent use across products in future surveillance. Sixth, the wide 95% confidence intervals for some e-cigarette use trends between 2017 and 2019 reflect two factors: prevalence in early waves was low, so large annual percentage changes corresponded to small absolute differences; and e-cigarette use was assessed in only a random subset of the sample in those years, which increased the variance of the estimates. As a result, APC estimates for small groups (e.g. dual use among Grade 8 students) should be interpreted with caution, since large percent changes can reflect small absolute differences when prevalence is low. Seventh, cigarette and e-cigarette use were self-reported; misreporting could introduce information bias and misclassification of use status, although the anonymous administration of the survey reduces social desirability pressures. Last, because the MTF survey samples students enrolled in school, the results may not extend to out-of-school youth.

## Conclusions

From 2017 to 2024, exclusive cigarette use and dual use of cigarettes and e-cigarettes among middle and high school students decreased. E-cigarette use rose until 2019–2020 and then declined or stabilized, remaining above 2017 levels among girls. Continued surveillance of sex- and grade-specific patterns of nicotine product use is warranted.
